# Clean Cut (adaptive, multimodal surgical infection prevention programme) for low‐resource settings: a prospective quality improvement study

**DOI:** 10.1002/bjs.11997

**Published:** 2020-09-21

**Authors:** J A Forrester, N Starr, T Negussie, D Schaps, M Adem, S Alemu, D Amenu, N Gebeyehu, T Habteyohannes, F Jiru, A Tesfaye, E Wayessa, R Chen, A Trickey, S Bitew, A Bekele, T G Weiser

**Affiliations:** 1 Department of Surgery, Stanford University, Stanford, USA; 2 Stanford‐Surgery Policy Improvement Research and Education Center, Department of Surgery, Stanford University, Palo Alto, USA; 3 Department of Surgery, University of California San Francisco, San Francisco California USA; 4 Duke University School of Medicine, Durham North Carolina USA; 5 Department of Surgery, School of Medicine, Addis Ababa University, Addis Ababa, Ethiopia; 6 Department of Surgery, Menelik II Referral Hospital, Addis Ababa, Ethiopia; 7 Lifebox Ethiopia, Addis Ababa, Ethiopia; 8 Departments of Surgery, , Jimma, Ethiopia; 9 Obstetrics and Gynaecology, School of Medicine, College of Health Sciences, Jimma, Ethiopia; 10 Department of Health Economics, Management, and Policy, Jimma University Medical Centre, Jimma, Ethiopia; 11 Quality Improvement Department, St Peter's Specialized Hospital, Fitche, Ethiopia; 12 Department of Surgery, St Peter's Specialized Hospital, Addis Ababa Gulele Subcity, Fitche, Ethiopia; 13 Fitche General Hospital, Fitche, Ethiopia; 14 Department of Clinical Surgery, University of Edinburgh, Edinburgh, UK

## Abstract

**Background:**

Clean Cut is an adaptive, multimodal programme to identify improvement opportunities and safety changes in surgery by enhancing outcomes surveillance, closing gaps in surgical infection prevention standards, and strengthening underlying processes of care. Surgical‐site infections (SSIs) are common in low‐income countries, so this study assessed a simple intervention to improve perioperative infection prevention practices in one.

**Methods:**

Clean Cut was implemented in five hospitals in Ethiopia from August 2016 to October 2018. Compliance data were collected from the operating room focused on six key perioperative infection prevention standards. Process‐mapping exercises were employed to understand barriers to compliance and identify locally driven improvement opportunities. Thirty‐day outcomes were recorded on patients for whom intraoperative compliance information had been collected.

**Results:**

Compliance data were collected from 2213 operations (374 at baseline and 1839 following process improvements) in 2202 patients. Follow‐up was completed in 2159 patients (98·0 per cent). At baseline, perioperative teams complied with a mean of only 2·9 of the six critical perioperative infection prevention standards; following process improvement changes, compliance rose to a mean of 4·5 (*P* < 0·001). The relative risk of surgical infections after Clean Cut implementation was 0·65 (95 per cent c.i. 0·43 to 0·99; *P* = 0·043). Improved compliance with standards reduced the risk of postoperative infection by 46 per cent (relative risk 0·54, 95 per cent c.i. 0·30 to 0·97, for adherence score 3–6 *versus* 0–2; *P =* 0·038).

**Conclusion:**

The Clean Cut programme improved infection prevention standards to reduce SSI without infrastructure expenses or resource investments.

## Teaser

Clean Cut is a multimodal, adaptive, checklist‐based infection prevention programme designed to improve compliance with six critical perioperative infection prevention practices. After introducing the programme at five hospitals in Ethiopia, compliance with critical infection prevention standards significantly improved and the relative risk of infection fell by 35 per cent.

keep it simple

## Resumen

**Antecedentes:**

‘*Clean Cut*’ (corte limpio) es un programa multimodal adaptable para identificar oportunidades de mejora y cambios en la seguridad a través de incrementar la vigilancia de los resultados, cerrar las brechas en los estándares de prevención de la infección quirúrgica y fortalecimiento de los procesos asistenciales subyacentes. Las infecciones del sitio quirúrgico (*surgical‐site infections*, SSI) son más frecuentes en los países de bajos ingresos, de modo que este estudio evaluó una intervención simple para mejorar la prevención de la infección perioperatoria en uno de estos países.

**Métodos:**

El programa ‘*Clean Cut*‘ se implementó en cinco hospitales de Etiopía desde agosto de 2016 a octubre de 2018. Los datos de cumplimiento se recogieron en el quirófano y se centraron en 6 estándares clave para la prevención de las infecciones perioperatorias. Se emplearon ejercicios de mapeo de procesos para comprender las barreras al cumplimiento e identificar oportunidades de mejora impulsadas localmente. Se registraron los resultados a los 30 días en los pacientes en los que se había recopilado información de cumplimiento intraoperatorio.

**Resultados:**

Se recogieron datos de cumplimiento de 2.213 operaciones (374 en situación basal y 1.839 tras las mejoras del proceso) en 2.202 pacientes. Se completó el seguimiento en 2.159 (98,0%) pacientes. En situación basal, los equipos perioperatorios solamente cumplieron en un promedio de 2,9 de 6 estándares críticos de prevención de la infección perioperatoria; tras los cambios del proceso de mejora, el cumplimiento llegó al 4,5 (*P* < 0,001). El riesgo relativo (RR) de infecciones quirúrgicas tras la implementación del programa “*Clean Cut*” fue de 0,65 (i.c. del 95% 0,43‐0,99; *P* = 0,043). La mejora del cumplimiento con los estándares redujo el riesgo de infección postoperatoria en un 46% (RR 0,45 para una puntuación de cumplimiento 3‐6 *versus* 0‐2; i.c. del 95% 0,30‐0,97; *P* = 0,038).

**Conclusion:**

El programa ‘*Clean Cut*’ mejoró los estándares de prevención para reducir las SSI sin infraestructura o asignación de recursos.

## Introduction

Postoperative infections are a leading cause of morbidity and mortality in surgical patients, particularly in low‐ and middle‐income countries. The WHO^1^ has assembled evidence‐based guidelines for surgical infection reduction. However, implementation of these guidelines is challenging, especially where resources, staff, and infrastructure are limited^2^^,^^3^. In high‐income settings, quality improvement efforts, such as the Surgical Care Improvement Program, focus on strengthening compliance with core process measures^4^. Such programmes are more difficult to implement in low‐income countries owing to human resource constraints, weak management practices and inadequate compliance measures; knowledge and training gaps are a further impediment to adopting best practices. As a result, infection rates are persistently higher in low‐resource settings, even when risk adjusting for differences in urgency and presentation; the GlobalSurg Collaborative^5^ reported that infection rates following gastrointestinal surgery were 23 per cent in low‐income countries (nearly twice that of high‐income countries)^5^, and the African Surgical Outcomes Study^6^ found that infections occur in 10 per cent of all patients undergoing surgery, of whom nearly one in ten die.

To address the combined challenges of high surgical infection rates and known gaps in perioperative practices, Lifebox^7^ – a non‐profit organization focused on improving surgical safety worldwide – established an infection reduction programme called Clean Cut to improve adherence to guidelines and reduce postoperative infection rates. Clean Cut is an adaptive, multimodal checklist‐based programme aimed at improving compliance with six critical perioperative infection prevention standards: appropriate skin and hand antisepsis, maintenance of a sterile field, instrument sterilization, appropriate prophylactic antibiotic administration, routine gauze counting, and routine use of the Surgical Safety Checklist (SSC)^8^. It is implemented by a locally led, multidisciplinary perioperative team that establishes a surveillance system for compliance with perioperative standards and surgical outcomes, conducts a process‐mapping exercise to determine gaps in perioperative processes and opportunities for improvement, and then facilitates the identification and implementation of locally driven solutions to improve upstream and supportive care routines. It leverages current concepts in implementation science, such as discrete implementation strategies, while also ensuring that specific implementation outcomes, such as acceptability and sustainability, are considered as the programme is adapted^9,10^. The authors hypothesized that this adaptive programme would promote compliance with these six critical infection prevention standards and lead to reductions in surgical infections.

## Methods

Clean Cut was introduced in five surgical referral hospitals in Ethiopia: Jimma University Specialized Hospital (JUSH) in Jimma; Tikur Anbessa Specialized Hospital (TASH), Menelik II Specialized Hospital (MII) – both affiliated with Addis Ababa University – and St Peter's Hospital in Addis Ababa; and Fitche Hospital in Oromia, Ethiopia. The first three are high‐volume tertiary teaching facilities, whereas St Peter's is a regional referral hospital and Fitche is a district hospital under the auspices of the Oromia Regional Health Bureau. Together they have a combined catchment population of over 25 million people. As the Ethiopian Federal Ministry of Health was preparing to launch a nationwide programme to improve access to safe surgical care^11^, the timing was opportune for such work. The programme was implemented between August 2016 and October 2018.

### Strategy

Clean Cut was designed to improve adherence to infection prevention standards through multidisciplinary team building, data collection and surveillance, and process mapping coupled with root cause analysis and identification of intervention opportunities. The aims of the programme were to assess process breakdowns, identify opportunities, and implement facility‐specific interventions to improve compliance with six surgical infection prevention standards, thereby reducing surgical infections, and was designed to be adaptive based on facility needs and circumstances. The standards targeted for improvement include: hand and surgical‐site skin antisepsis; maintenance of the sterile field by ensuring integrity and sterility of gowns, drapes and gloves; appropriate instrument decontamination and sterilization; appropriate timing and selection of prophylactic antibiotics; routine surgical gauze counting; and routine use of the SSC. Three of these standards are embedded within the checklist itself: appropriate timing of antibiotics within 60 min of skin incision, confirmation of instrument sterility and routine swab counting. Two others – appropriate hand and surgical‐site preparation, and use of sterile gowns, drapes and gloves – are fundamental tenets of surgical antisepsis. As use of the SSC is now itself a standard of care, it was included as a means of promoting communication among the perioperative team and reinforcing the other five practices. The definitions and rationale for these six specific standards have been described previously^12^.

The programme follows a typical plan–do–study–act cycle by which locally driven interventions were identified and implemented, and adherence to process reassessed continually for improvements. To achieve these goals, Clean Cut used three sequential steps: team building through the introduction and local modification of the SSC with clinical stakeholders (administration, surgeons, anaesthesia providers, operating theatre nursing staff); ongoing assessment of compliance with perioperative infection prevention standards and patient outcomes using trained data collectors; and process improvement through a locally led process‐mapping exercise^13^ and feedback cycle that included stakeholder meetings to review compliance to infection prevention standards and patient outcomes, brainstorming of solutions and prioritization of interventions. A surgical resident supported implementation by organizing team meetings once or twice per month at each of the hospitals, guiding initial modification of the checklist, conducting training for staff to ensure accurate data collection, leading process‐mapping exercises, and facilitating the review of surveillance and process‐mapping information to identify opportunities for improvement.

A 4–6‐week lead‐in period focusing on team building and assessments of process compliance and surgical outcomes, as well as the process‐mapping work, was planned. At the 4–6‐week mark the teams met and, using the compliance information collected, identified opportunities for improvement with specific plans for process changes. Teams met every few months and again at the end of the 6‐month programme to review progress and opportunities for further improvement. The entire programme was implemented over 6 months at each hospital, with ongoing assessments of process compliance. Power calculations and details of the implementation timeline are available in *Appendix S1* and *Fig. S1* (supporting information).

### Intraoperative adherence data collection

Observed perioperative practices were recorded on a previously validated, standardized paper form by data collectors (operating theatre nurses and nurse anaesthetists), who were trained in its use and the definitions and mechanism for collection^14^. At JUSH, any patient undergoing surgical intervention in the main and obstetric operating rooms was included, regardless of age, sex or diagnosis. As a large volume of subspecialty operations is performed at TASH and MII, observations were limited to specific operating theatres performing gastrointestinal surgery, emergency general surgery or obstetric operations, regardless of age, sex or diagnosis. At St Peter's, all patients undergoing surgery in the general surgery theatres were eligible. In Fitche, every operation performed in one of its two operating theatres was eligible for inclusion. Detailed data collection methods for perioperative adherence have been described previously^13^. Although information was collected on all antibiotic administration, for compliance calculations only those given as prophylaxis were considered; antibiotics administered as treatment based on wound class and preoperative diagnosis were not included in the analysis.

### Patient outcomes data collection

Postoperative surveillance was carried out on all patients who underwent observation of perioperative adherence to the six standards. The primary outcome was surgical‐site infection (SSI); secondary outcomes included duration of hospital stay and mortality. SSIs were classified as superficial, deep or organ space according to Centers for Disease Control and Prevention (CDC) definitions^15^. To operationalize the SSI definition, standardize capture and decrease the potential for misclassification, SSI was defined by pus draining from the wound, previously closed wound now opened (intentionally or unintentionally) and wound with foul smell.

**Fig. 1:**
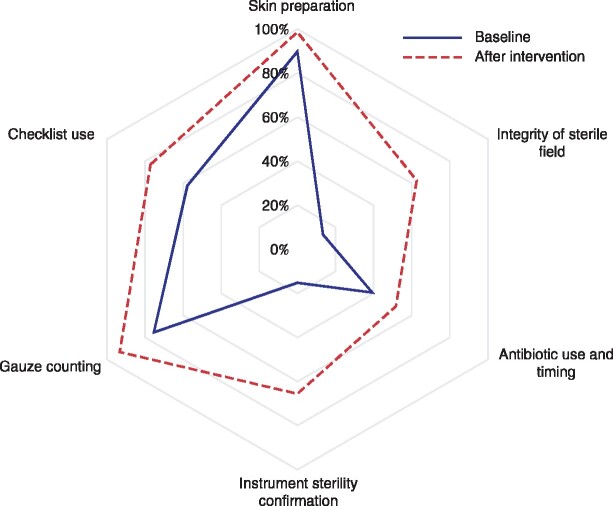
Radar plot of adherence to each Clean Cut standards category at baseline and after process improvement interventions

Perfect compliance is defined by the outer edge of the plot. *P* < 0·001 for all improvement processes (Fisher's exact test).

For the initial piloting of the programme at Jimma, surveillance data were collected by review of the written medical record at discharge by a visiting surgical fellow and trained data collectors. Given the gross underestimation of infectious complications based on chart review alone^12^, the surveillance methods were modified for the other four sites, where inpatient surveillance was conducted by direct observation of the surgical wounds and brief review of the written medical chart by a group of ward nurses from postoperative day 1 until discharge. As the majority of surgical infectious complications typically occur after discharge^16^, telephone follow‐up was undertaken 30 days after surgery in the local language for outpatient surveillance using the wound definitions described above. For all patients with suspicion of a complication (such as patient undergoing a clean operation on extended antibiotic treatment; reoperation; death), the written medical charts were reviewed manually by the visiting surgical fellows to assess accuracy and evaluate discrepancies.

### Identification of process breakdowns and opportunities

Breakdowns in the perioperative processes of care frequently lead to failure in compliance with one or more of the infection prevention standards. Process breakdowns and potential opportunities for improvement were identified using process mapping, a technique adapted from manufacturing involving preparation of diagrams of activities, tasks and decisions within a work flow in order to understand and subsequently improve the overall process^17^. This specific technique has been described previously for the Clean Cut programme^13^. The frequency with which certain process failures occurred was also recorded on the process maps.

### Facility feedback and implementation

Quantitative adherence to the Clean Cut perioperative infection prevention standards was compiled over a 4–6‐week baseline period. The degree of adherence and visual process map for each infection prevention standard were delivered to the local stakeholders through individual and focus group meetings, as described previously^13^. Up‐to‐date patient outcome data were included in these meetings to provide context. After development and prioritization of facility‐specific interventions, two subsequent stakeholder meetings were held during the course of the 6‐month programme to assess interventions and outcomes. Identified interventions included: improving the use of alcohol hand rub following surgical hand scrubbing; establishing a procedure to mend holes in gowns and drapes; training operating room cleaning staff on appropriate decontamination and packaging of instruments, including use of sterility indicator tape in instrument trays; establishing a standard for gauze counting; and verbal use of the checklist at critical moments before induction of anaesthesia and skin incision.

### Data analysis

As this was a quality improvement initiative to improve perioperative practices and did not introduce new clinical methods or involve any direct risk to patients, there were no exclusion criteria; all patients undergoing surgery in the operating theatres selected for evaluation were eligible and patient consent was not obtained. Institutional review boards at Stanford University, the College of Health Sciences at Jimma University and the College of Health Sciences at Addis Ababa University approved the study; approval was also obtained from appropriate administrative bodies at each hospital. Neither patients nor the public were involved in designing this study.

Demographic and clinical characteristics of patients in the baseline phase and after the intervention were compared using χ^2^ tests for categorical variables and Student's *t* test for age as a continuous variable. Adherence to the six standards at baseline compared with after intervention was evaluated in univariable analyses using χ^2^ tests. The relative risk of infection after implementation of the programme was calculated using modified Poisson regression analysis with a robust error variance^18^, controlling for age, sex, urgency, wound class, type of operation and hospital modelled as fixed effects. The relative risk of infection was also assessed based on the degree of adherence to the standards of interest using a similar model adjusted for the same co‐variables. Sensitivity analyses were undertaken using multivariable logistic regression models, controlling for the same factors, to assess the relative risk of infection during the postintervention phase as well as with increased adherence to infection prevention standards; these regression models were also computed using log‐binomial regression, as well as mixed‐effects hierarchical regression with a random effect by hospital (*Appendix S1*, supporting information) and other co‐variables modelled as fixed effects. All analyses were preplanned, statistical significance was assessed at the level α = 0·05, and all tests were two‐sided. SAS® version 9.4 (SAS Institute, Cary, North Carolina, USA) was used for statistical analysis.

## Results

Compliance data were collected prospectively from 2213 operations in 2202 patients, 374 during baseline assessment and 1839 after implementation. Follow‐up outcomes were complete for 2159 patients (98·0 per cent). Demographics of the patients in the baseline and postintervention cohorts are shown in *Table* *1*. There were proportionally more emergency and contaminated operations after the intervention, but age, sex and the variety of operations were well matched.

**Table 1:** Patient demographics and operations before and after process improvement interventions

	Baseline (*n* = 374)	After implementation (*n* = 1839)	*P* ^**^
**Age (years)** *	35·7(16·4)	34·0(14·5)	0·061^††^
≤ 25	108 (29·0)	572 (31·2)	0·125
26–30	78 (21·0)	449 (24·5)	
31–40	82 (22·0)	396 (21·6)	
≥ 41	104 (28·0)	416 (22·7)	
Missing	2	6	
**Sex ratio (M** : **F)**	250 : 124	1321 : 518	0·053
**Urgency**			< 0·001
Elective	235 (62·8)	892 (48·5)	
Emergency	139 (37·2)	947 (51·5)	
**Type of operation**			< 0·001
Orthopaedic^†^	6 (1·6)	31 (1·7)	
Soft tissue^‡^	20 (5·3)	70 (3·8)	
Ear, nose and throat^§^	42 (11·2)	128 (7·0)	
Gynaecological^¶^	15 (4·0)	46 (2·5)	
Vascular	6 (1·6)	36 (2·0)	
Appendicectomy	32 (8·6)	183 (10·0)	
Cholecystectomy	12 (3·2)	28 (1·5)	
Colorectal	21 (5·6)	104 (5·7)	
Caesarean	107 (28·6)	737 (40·1)	
Hernia	9 (2·4)	58 (3·2)	
Hysterectomy	16 (4·3)	39 (2·1)	
Gastrointestinal/laparotomy^#^	54 (14·4)	307 (16·7)	
Urological	34 (9·1)	71 (3·9)	
Missing	0	1	
**Wound classification**			0·100
Clean	68 (18·2)	316 (17·2)	
Clean contaminated	254 (67·9)	1185 (64·5)	
Contaminated	40 (10·7)	288 (15·7)	
Dirty	12 (3·2)	49 (2·7)	
Missing	0	1	

Values in parentheses are percentages unless indicated otherwise;

* values are mean(s.d.).

† Includes extremity amputations;

‡ includes skin, breast and external anal procedures;

§ head and neck operations, including thyroid;

¶ includes operations for ectopic pregnancy;

# gastrointestinal and other intra‐abdominal operations via a laparotomy.

** χ^2^ test, except

†† Student's *t* test with Satterthwaite method.

Adherence to each of the infection prevention standards significantly improved after process mapping and quality improvement interventions (*Fig*. *1*). All standards had major gaps and weaknesses, as each had multiple components of compliance and frequently one or two of these were critically deficient. However, adherence to each standard increased as processes were strengthened and component parts of full compliance were enhanced (*Table* *2*). For example, for assurance of instrument sterility, both the presence of sterile indicators and the absence of condensation and fluid within the trays are critical to ensuring appropriate sterility of the instrument tray. However, in the baseline period only 133 of 374 surgical trays had sterility indicators located in the tray, and 123 had condensation (indicative of a poor heating cycle of the steam autoclave). Refining the process by which sterility indicators are included in the instrument trays, gowns and drapes, and adjusting the autoclave, improved compliance with instrument sterility standards more than fourfold, and gown and drape sterility standards almost fivefold (*Table* *2*).

**Table 2:** Adherence to Clean Cut surgical infection prevention standards at baseline and after process improvement interventions

	Baseline (*n* = 374)	After implementation (*n* = 1839)	*P* ^*^
**Skin preparation compliance**			
Surgeon hand scrub completed	340 (90·9)	1824 (99·2)	< 0·001
Surgical site skin preparation completed	370 (98·9)	1828 (99·4)	0·311
Total compliance	336 (89·8)	1814 (98·6)	< 0·001
**Sterile field compliance**			
New surgical gloves used by surgeon	371 (99·2)	1832 (99·6)	0·268
Sterility indicator present inside gown and drape pack	52 (13·9)	1209 (65·7)	< 0·001
If so, had colour changed indicating sterility	51 (98·1)	1188 (98·3)	0·920
Dry gowns and drapes used	251 (67·1)	1819 (98·9)	< 0·001
Gowns without holes or tears	366 (97·9)	1815 (98·7)	0·218
Drapes without holes or tears	366 (97·9)	1832 (99·6)	< 0·001
Total compliance	50 (13·4)	1151 (62·6)	< 0·001
**Instrument sterility compliance**			
Sterility indicator present inside instrument tray	133 (35·6)	1258 (68·4)	< 0·001
If so, had colour changed indicating sterility	132 (99·2)	1240 (98·6)	0·521
Inside of the instrument tray dry	251 (67·1)	1801 (97·9)	< 0·001
Total compliance	57 (15·2)	1207 (65·6)	< 0·001
**Antibiotic administration compliance**			
Antibiotics given before surgery	322 (86·1)	1706 (92·8)	< 0·001
Antibiotics given in operating room	110 (29·4)	877 (47·7)	< 0·001
Total compliance	147 (39·3)	951 (51·7)	< 0·001
**Gauze count compliance**			
Gauze count before operation	301 (80·5)	1756 (95·5)	< 0·001
Gauze count after operation	294 (78·6)	1749 (95·1)	< 0·001
Total compliance	282 (75·4)	1716 (93·3)	< 0·001
**Checklist compliance**			
Procedure announced before the start of the operation	304 (81·3)	1716 (93·3)	< 0·001
Time out/team introductions performed	231 (61·8)	1457 (79·2)	< 0·001
Estimated blood loss stated aloud	319 (85·3)	1678 (91·2)	< 0·001
Total compliance	216 (57·8)	1417 (77·1)	< 0·001

Values in parentheses are percentages. Total compliance includes full compliance with each of the processes within the category.

* χ^2^ test.

At baseline, perioperative teams complied with only a mean of 2·9 of the six critical perioperative infection prevention standards; after implementation of process improvement changes, compliance rose to a mean of 4·5 (*P* < 0·001) (*Table* *3*). This improvement in compliance resulted in a non‐significant reduction in surgical infections from 7·4 to 5·8 per cent (*P =* 0·246) on univariable analysis. In multivariable analysis controlling for sex, age, urgency, wound class, type of operation and facility, the relative risk of infection following Clean Cut implementation was 0·65 (95 per cent c.i. 0·43 to 0·99; *P =* 0·043) (*Table* *4*). Improved compliance was significantly associated with reductions in postoperative infections. Comparing operations with low compliance (2 or fewer critical standards) *versus* those with high compliance (3 or more), the relative risk of infection dropped by 46 per cent (relative risk 0·54, 0·30 to 0·97; *P =* 0·038) (*Table* *5*). Sensitivity analyses showed the point estimate results to be robust to several different modelling strategies (*Appendix S1*, supporting information). Duration of hospital stay remained unchanged, and owing to low numbers of deaths (20 in total) it was not possible to use multivariable modelling to assess the relative risk of death after programme implementation.

**Table 3:** Overall adherence score at baseline and after process improvement interventions

	Adherence score (out of 6)			
	Baseline (*n* = 374)	After implementation (*n* = 1839)	Difference	*P*
Mean (95% c.i.)	2·9 (2·8, 3·1)	4·5 (4·4, 4·5)	1·6 (1·4, 1·7)	< 0·001*
Median (i.q.r.)	3 (2–4)	5 (4–5)		< 0·001^†^

* Student's *t* test with Satterthwaite method;

^†^Wilcoxon rank‐sum test.

**Table 4:** Relative risk of postoperative infection based on timing of process improvements, after adjusting for sex, age, urgency, wound class and hospital using modified robust Poisson regression

	Relative risk	*P*
**Infection**		
Baseline	1·00 (reference)	
After implementation	0·65 (0·43, 0·99)	0·043
**Sex**		
F	1·00 (reference)	
M	0·83 (0·52, 1·32)	0·429
**Age (years)**		
≤ 25	1·00 (reference)	
26–30	0·72 (0·45, 1·15)	0·169
31–40	0·81 (0·50, 1·30)	0·382
≥ 41	0·82 (0·46, 1·47)	0·511
**Urgency**		
Elective	1·00 (reference)	
Emergency	1·10 (0·70, 1·74)	0·683
**Wound class**		
Clean	1·00 (reference)	
Clean contaminated	0·38 (0·08, 1·68)	0·202
Contaminated	0·84 (0·18, 3·87)	0·827
Dirty	2·01 (0·44, 9·22)	0·367
**Type of operation**		
Ear, nose and throat	1·00 (reference)	
Orthopaedic	4·44 (0·63, 31·19)	0·134
Soft tissue	3·72 (0·92, 15·09)	0·066
Gynaecological	4·36 (0·62, 30·44)	0·138
Vascular	1·22 (0·12, 12·27)	0·867
Appendicectomy	4·73 (0·76, 29·63)	0·097
Cholecystectomy	9·89 (1·20, 81·43)	0·033
Colorectal	10·93 (1·76, 67·92)	0·010
Caesarean	11·09 (1·82, 67·74)	0·009
Hernia	2·31 (0·40, 13·34)	0·348
Hysterectomy	12·09 (1·63, 89·57)	0·015
Gastrointestinal/laparotomy	5·48 (0·96, 31·31)	0·056
Urological	3·92 (0·30, 50·67)	0·295

Values in parentheses are 95 per cent confidence intervals. Relative risks by hospital are not shown.

**Table 5:** Relative risk of postoperative infection based on adherence to six critical standards in infection prevention, after adjusting for sex, age, urgency, wound class and hospital using modified robust Poisson regression

	Relative risk	*P*
**Adherence score**		
0–2	1·00 (reference)	
3–6	0·54 (0·30, 0·97)	0·038
**Sex**		
F	1·00 (reference)	
M	0·82 (0·51, 1·32)	0·414
**Age (years)**		
≤ 25	1·00 (reference)	
26–30	0·72 (0·45, 1·14)	0·162
31–40	0·82 (0·51, 1·32)	0·418
≥ 41	0·80 (0·45, 1·44)	0·460
**Urgency**		
Elective	1·00 (reference)	
Emergency	1·08 (0·68, 1·72)	0·740
**Wound class**		
Clean	1·00 (reference)	
Clean contaminated	0·40 (0·09, 1·83)	0·237
Contaminated	0·87 (0·19, 4·07)	0·860
Dirty	2·10 (0·45, 9·77)	0·345
**Type of operation**		
Ear, nose and throat	1·00 (reference)	
Orthopaedic	4·38 (0·60, 32·24)	0·147
Soft tissue	3·47 (0·85, 14·14)	0·083
Gynaecological	3·99 (0·59, 26·88)	0·155
Vascular	1·14 (0·11, 11·33)	0·912
Appendicectomy	4·76 (0·74, 30·69)	0·101
Cholecystectomy	9·37 (1·10, 79·70)	0·041
Colorectal	11·08 (1·74, 70·58)	0·011
Caesarean	9·94 (1·58, 62·72)	0·015
Hernia	2·33 (0·40, 13·46)	0·343
Hysterectomy	11·08 (1·45, 84·72)	0·021
Gastrointestinal/laparotomy	5·41 (0·92, 31·90)	0·062
Urological	4·01 (0·31, 51·93)	0·288

Values in parentheses are 95 per cent confidence intervals. The analysis was based on 2159 procedures. Relative risks by hospital are not shown.

## Discussion

Using an adaptive, multimodal intervention, five hospitals in Ethiopia with substantial resource limitations were able to significantly improve compliance with six critical standards of infection prevention and reduced the risk of postoperative infections by 35 per cent; mortality and duration of hospital stay remained unchanged. Appropriate implementation was accomplished through a combination of collaborative, cross‐disciplinary efforts coupled with surveillance of processes and outcomes, and a process‐mapping exercise to identify gaps and opportunities for improvement. The SSC was used both as an audit tool to identify compliance gaps and as a safety tool to leverage improvements in perioperative care through a combination of process mapping and teamwork.

Although use of the WHO SSC has spread widely, implementation and compliance are challenging^19^. Evidence suggests that facilities demonstrably able to implement the checklist appropriately can reduce complications^20^^,^^21^, presumably owing to the ability of those facilities to comply with the standards embedded in the checklist and to meet the spirit of the checklist as a tool to improve communication among perioperative team members. In low‐income countries, compliance with the checklist is hindered by severe resource constraints, staff shortages, weak processes and limited infrastructure^22^^,^^23^. Adoption of the checklist can be seen as impractical, particularly when resource constraints prohibit compliance with many of its critical elements. However, many safety checks are based on communication and confirmation of processes that are part of standard operating procedure, so checklist use can be implemented even in settings with compliance challenges^24^. Indeed, compliance challenges are present universally, regardless of setting, and teams must work to overcome them when implementing the checklist.

Although barriers to surgical improvement and safety in low‐income countries are frequently ascribed to infrastructural limitations, the mechanisms by which safety tools like the checklist are introduced appear to matter a great deal^25^^,^^26^. Clean Cut aims to strengthen underlying processes of care that cannot be corrected easily with the introduction of the checklist alone. As a quality improvement programme, it is supported by three fundamental and interconnected strategies: the building of a multidisciplinary team; appropriate surveillance of compliance with standards and outcomes of care; and process mapping coupled with a mechanism to identify opportunities for improving compliance with the six critical standards of surgical infection prevention. Process mapping allowed the team to identify gaps in workflow, deficiencies in standard operating procedures, poor or inadequate training and misperceptions of appropriate infection prevention practices, and weaknesses in management oversight. The data collected enumerating the lack of compliance with standards were a powerful motivator to establish an improvement programme. Finally, the multidisciplinary nature of the team allowed each member to leverage their respective position to enact changes in workflow within their sphere of influence. Furthermore, it allowed multiple disciplines to collectively lobby for attention and resources that had not previously been identified or agreed on. Clean Cut also used implementation strategies that were often not readily available to providers given the way facilities and human resources were managed and organized before the programme.

A limitation of this study is its pre–post design, and lack of involvement with patient advocates and the general public during planning and delivery. Similar to many surgical safety checklist and other quality improvement studies^27^, this study did not randomize patients or hospitals. Checklists are considered a standard of care in many countries, and improved checklist use is a priority for the Federal Ministry of Health of Ethiopia^11^; as such, randomization was impracticable as part of the strategy was to improve appropriate compliance with the checklist. Data collectors were not specifically blinded to the intervention, but neither were they necessarily aware of when specific interventions were made within the facility, as many improvements were undertaken outside of the operating theatre (such as how antibiotics were delivered to patients or how sterility of instruments was assured). Furthermore, although oversight was provided by the senior author, data analysis was undertaken by a separate team not involved in programme implementation. Data collection was challenging, and obtaining 30‐day follow‐up information was limited by the ability to reach patients and families by telephone; despite this, it was possible to follow up 98·0 per cent of patients undergoing surgery, a much higher proportion than originally anticipated. An additional unexpected finding was the satisfaction the nursing staff expressed at being able to contact patients after discharge^23^. The observed infection rates were lower than has been reported previously in these settings, although still fairly high overall. The diagnosis of surgical infection was modified from the CDC definition, and antibiotics are commonly used empirically after surgery without regard to the actual presence of infection. Imaging modalities and other ancillary testing such as laboratory blood work was not readily available; definitions were therefore based on clearly defined, easily observable objective signs of surgical infection (such as pus, reopening of a closed wound, or foul smell emanating from the wound). This may have limited the ability to capture more subtle wound infections, but as these definitions were applied consistently during the study the findings are still valid. Improvements in the ability to detect infections were noted as the study progressed, but this would be expected to bias the study against findings of improvement. Furthermore, this study was not powered to detect significant improvements at individual facilities and, in agreement with each facility, all results are reported in aggregate owing to reputation considerations. However, each site was able to improve compliance with standards to some degree, indicating an ability to enact positive changes to the perioperative routine (*Appendix S1*, supporting information). A Hawthorne effect rather than the programme itself might also explain improvements in both compliance and outcomes, although it is a phenomenon that those implementing quality improvement programmes leverage to their advantage. Finally, despite intensive efforts, it was rarely possible to attain full compliance with all six standards. This attests to the ongoing challenges in Ethiopia and other resource‐constrained environments in fully implementing safety programmes and improving care processes in facilities with multiple organizational challenges.

Implementation of Clean Cut required the concerted efforts of a number of practitioners and administrators not typically used to working together. It also required the ongoing support of a programme manager and surgical research fellow to help coordinate the work across facilities, and leverage support within them and with the health ministry. As much of the data did not exist separately from the programme, the hiring of data collectors was seen as critical. For all these reasons, long‐term sustainability is not yet proven. Furthermore, the actual implementation costs are unclear. Start‐up costs to develop, adapt and assess the programme were substantial, but subsequent work has required sequentially fewer resources; new‐site onboarding and ongoing maintenance costs are under investigation. However, the programme now has the support of the Federal Ministry of Health, and Clean Cut is being rolled out in a number of new facilities using staff familiar with the programme to encourage the new teams and to drive training, organization of workflow and high‐quality data collection. The authors continue to support the efforts in Ethiopia and anticipate bringing Clean Cut to new settings in the coming years. A robust, large‐scale experimental study, such as a pragmatic stepped‐wedge cluster‐randomized trial, would be ideally suited to test the intervention more rigorously, and allow closer observation of the features and strategies that lead to successful implementation^27^.

## Supplementary Material

znaa187_Supplementary_Data
