# Supplementary material for: Clean Cut (adaptive, multimodal surgical infection prevention programme) for low‐resource settings: a prospective quality improvement study
Source: Br J Surg. 2020 Sep 21;108(6):727–34. doi: 10.1002/bjs.11997 (PMC10364890; doi:10.1002/bjs.11997)
Supplement: znaa187_Supplementary_Data [file znaa187_supplementary_data.docx]

**BJS11997**

**Clean Cut (adaptive, multimodal surgical infection prevention programme) for low-resource settings: a prospective quality improvement study**

J. A. Forrester, N. Starr, T, Negussie, D. Schaps, M. Adem, S. Alemu, D. Amenu, N. Gebeyehu, T. Habteyohannes, F. Jiru, A. Tesfaye, E. Wayessa, R. Chen, A. Trickey, S. Bitew, A. Bekele and T. G. Weiser

**Appendix S1**

Power calculations assumed a baseline postoperative infection rate of 10%, an effect size equivalent to a 50% reduction in infections, and a 5:1 ratio of patients in the post-implementation vs baseline period. This gave a total sample size of 1416, with 236 in the baseline period and 1180 in the post-implementation period. We aimed approximately 20 patients per week per facility; approximately 400 in the baseline and 1700 following implementation, with a total of 2080 patients.

At each facility, the program was introduced during the first week which included gathering the perioperative team involved in the work and working through the Surgical Safety Checklist to make modifications to meet local needs. The second week was spent training data collectors to capture both compliance information as well as surgical outcomes. During this week data collection commenced in a mentored fashion to ensure accuracy. Process mapping exercises were carried out over a number of weeks until all six processes were mapped out at the facility. At the end of 6-8 weeks, these activities were completed and the team met to review the data and the process maps, and identify changes that could be made to improve compliance (thick arrow). Monthly meetings were held to review compliance data and identify ongoing opportunities for improvement. Compliance data capture continued for 6 months total, while outcomes data collection continued for an additional month to ensure 30-day follow up.

**Fig. S1:**

Collect surgical outcomes data

Collect process compliance data

Gather perioperative team, introduce and modify the Checklist

Train data collectors

Conduct process mapping exercises

Program introduction

Initial data review and process improvement planning

Interim feedback

Final feedback

End data collection

Interim feedback

Since there were significant differences between the baseline and post-implementation groups, we performed a robust modified Poisson regression to assess the relative risk of infection. We performed several different multivariable regression models to corroborate our findings: we used logistic regression to determine the odds ratio of surgical infection before and after implementation, as well as log-binomial regression as an additional statistical strategy. For all initial analyses, the hospital was considered as a fixed effect in the models; other covariates used in our regression models were age, sex, urgency, wound class, and type of operation. We repeated these analyses using mixed-effects models with random intercepts for hospitals to determine whether our point estimates would hold steady, understanding that this would widen our confidence intervals. Our results for fixed vs mixed effects models are as follows:

**Table S1:**

|  | **Fixed Effects** | | | | | | **Random Effects** | | | | | |
| --- | --- | --- | --- | --- | --- | --- | --- | --- | --- | --- | --- | --- |
|  | **Logistic Regression** | | **Modified Robust Poisson** | | **Log Binomial** | | **Logistic Regression** | | **Modified Robust Poisson** | | **Log Binomial** | |
| **Factor** | **SSI (N=2159)**  **OR (95% CI)** | **p-value** | **SSI (N=2159)**  **RR (95% CI)** | **p-value** | **SSI (N=2159)**  **RR (95% CI)** | **p-value** | **SSI (N=2159)**  **OR (95% CI)** | **p-value** | **SSI (N=2159)**  **RR (95% CI)** | **p-value** | **SSI (N=2159)**  **RR (95% CI)** | **p-value** |
| **Pre/Post** |  |  |  |  |  |  |  |  |  |  |  |  |
| Pre | Reference |  | Reference |  | Reference |  | Reference |  | Reference |  | Reference |  |
| Post | 0.63 (0.39, 0.997) | 0.049 | 0.65 (0.43, 0.99) | 0.043 | 0.66 (0.44, 0.99) | 0.047 | 0.64 (0.40, 1.01) | 0.055 | 0.66 (0.37, 1.18) | 0.16 | 0.67 (0.44, 1.01) | 0.055 |
| **Sex** |  |  |  |  |  |  |  |  |  |  |  |  |
| Female | Reference |  | Reference |  | Reference |  | Reference |  | Reference |  | Reference |  |
| Male | 0.80 (0.47, 1.39) | 0.44 | 0.83 (0.52, 1.32) | 0.43 | 0.83 (0.52, 1.34) | 0.45 | 0.80 (0.46, 1.38) | 0.42 | 0.82 (0.53, 1.28) | 0.38 | 0.83 (0.51, 1.33) | 0.43 |
| **Age** |  |  |  |  |  |  |  |  |  |  |  |  |
| <=25 | Reference |  | Reference |  | Reference |  | Reference |  | Reference |  | Reference |  |
| 26-30 | 0.70 (0.43, 1.16) | 0.17 | 0.72 (0.45, 1.15) | 0.17 | 0.70 (0.44, 1.10) | 0.13 | 0.72 (0.44, 1.18) | 0.19 | 0.74 (0.45, 1.20) | 0.22 | 0.72 (0.45, 1.13) | 0.16 |
| 31-40 | 0.80 (0.48, 1.34) | 0.39 | 0.81 (0.50, 1.30) | 0.38 | 0.80 (0.50, 1.27) | 0.34 | 0.82 (0.49, 1.37) | 0.45 | 0.83 (0.62, 1.11) | 0.21 | 0.82 (0.51, 1.31) | 0.40 |
| >=41 | 0.81 (0.44, 1.50) | 0.51 | 0.82 (0.46, 1.47) | 0.51 | 0.82 (0.48, 1.42) | 0.48 | 0.83 (0.45, 1.53) | 0.56 | 0.84 (0.63, 1.13) | 0.26 | 0.84 (0.48, 1.44) | 0.52 |
| **Urgency** |  |  |  |  |  |  |  |  |  |  |  |  |
| Elective | Reference |  | Reference |  | Reference |  | Reference |  | Reference |  | Reference |  |
| Emergency | 1.12 (0.67, 1.87) | 0.66 | 1.10 (0.70, 1.74) | 0.68 | 1.07 (0.68, 1.68) | 0.77 | 1.07 (0.64, 1.79) | 0.79 | 1.05 (0.75, 1.47) | 0.76 | 1.03 (0.65, 1.63) | 0.90 |
| **Wound Class** |  |  |  |  |  |  |  |  |  |  |  |  |
| Clean | Reference |  | Reference |  | Reference |  | Reference |  | Reference |  | Reference |  |
| Clean Contaminated | 0.34 (0.09, 1.37) | 0.13 | 0.38 (0.08, 1.68) | 0.20 | 0.40 (0.11, 1.45) | 0.16 | 0.34 (0.09, 1.33) | 0.12 | 0.37 (0.09, 1.62) | 0.19 | 0.40 (0.11, 1.41) | 0.15 |
| Contaminated | 0.82 (0.19, 3.50) | 0.78 | 0.84 (0.18, 3.87) | 0.83 | 0.88 (0.23, 3.38) | 0.85 | 0.83 (0.20, 3.49) | 0.79 | 0.85 (0.30, 2.46) | 0.77 | 0.90 (0.24, 3.34) | 0.87 |
| Dirty | 2.37 (0.55, 10.29) | 0.25 | 2.01 (0.44, 9.22) | 0.37 | 2.13 (0.56, 8.16) | 0.27 | 2.36 (0.55, 10.06) | 0.25 | 2.02 (0.55, 7.39) | 0.29 | 2.15 (0.58, 7.95) | 0.25 |
| **Hospital** |  |  |  |  |  |  |  |  |  |  |  |  |
| 1 | Reference |  | Reference |  | Reference |  |  |  |  |  |  |  |
| 2 | 0.69 (0.32, 1.52) | 0.36 | 0.70 (0.33, 1.48) | 0.35 | 0.71 (0.34, 1.47) | 0.35 |  |  |  |  |  |  |
| 3 | 2.10 (1.02, 4.30) | 0.043 | 1.95 (1.03, 3.68) | 0.041 | 2.07 (1.10, 3.90) | 0.02 |  |  |  |  |  |  |
| 4 | 1.24 (0.58, 2.65) | 0.59 | 1.21 (0.60, 2.44) | 0.60 | 1.23 (0.61, 2.47) | 0.56 |  |  |  |  |  |  |
| 5 | 1.99 (1.03, 3.85) | 0.042 | 1.86 (1.05, 3.28) | 0.033 | 1.86 (1.03, 3.34) | 0.038 |  |  |  |  |  |  |
| **Type of operation** |  |  |  |  |  |  |  |  |  |  |  |  |
| ENT | Reference |  | Reference |  | Reference |  | Reference |  | Reference |  | Reference |  |
| Orthopedic | 4.82 (0.67, 34.88) | 0.12 | 4.44 (0.63, 31.19) | 0.13 | 4.27 (0.66, 27.77) | 0.13 | 4.31 (0.61, 30.50) | 0.14 | 3.99 (1.96, 8.14) | <0.001 | 3.89 (0.60, 25.15) | 0.15 |
| Soft tissue | 3.97 (0.89, 17.65) | 0.071 | 3.72 (0.92, 15.09) | 0.066 | 3.77 (0.90, 15.73) | 0.068 | 3.72 (0.84, 16.41) | 0.083 | 3.50 (2.15, 5.70) | <0.001 | 3.57 (0.86, 14.82) | 0.080 |
| Gynecologic | 4.82 (0.64, 36.21) | 0.13 | 4.36 (0.62, 30.44) | 0.14 | 4.32 (0.63, 29.78) | 0.14 | 4.32 (0.59, 31.64) | 0.15 | 3.93 (1.69, 9.11) | 0.002 | 3.89 (0.58, 26.34) | 0.16 |
| Vascular | 1.22 (0.12, 12.55) | 0.87 | 1.22 (0.12, 12.27) | 0.87 | 1.25 (0.13, 12.15) | 0.85 | 1.19 (0.12, 12.16) | 0.88 | 1.20 (0.18, 7.83) | 0.85 | 1.22 (0.13, 11.71) | 0.86 |
| Appendectomy | 5.15 (0.78, 33.93) | 0.088 | 4.73 (0.76, 29.63) | 0.10 | 4.63 (0.79, 27.28) | 0.090 | 5.43 (0.84, 35.24) | 0.076 | 4.97 (0.79, 31.53) | 0.089 | 4.85 (0.84, 27.80) | 0.077 |
| Cholecystectomy | 11.83 (1.35, 103.56) | 0.026 | 9.89 (1.20, 81.43) | 0.033 | 8.79 (1.19, 65.10) | 0.033 | 11.30 (1.33, 96.26) | 0.027 | 9.54 (1.85, 49.08) | 0.007 | 8.50 (1.14, 63.11) | 0.036 |
| Colorectal | 13.62 (2.12, 87.48) | 0.006 | 10.93 (1.76, 67.92) | 0.010 | 10.03 (1.77, 56.91) | 0.009 | 13.70 (2.16, 86.92) | 0.006 | 11.05 (3.33, 36.68) | <0.001 | 10.08 (1.80, 56.33) | 0.009 |
| Cesarean | 13.10 (1.94, 88.31) | 0.008 | 11.09 (1.82, 67.74) | 0.009 | 11.04 (1.85, 65.92) | 0.008 | 12.38 (1.91, 80.47) | 0.008 | 10.55 (2.32, 47.96) | 0.002 | 10.38 (1.79, 60.23) | 0.009 |
| Hernia | 2.42 (0.38, 15.46) | 0.35 | 2.31 (0.40, 13.34) | 0.35 | 2.28 (0.38, 13.63) | 0.37 | 2.35 (0.37, 15.01) | 0.37 | 2.25 (0.73, 6.93) | 0.16 | 2.23 (0.37, 13.42) | 0.38 |
| Hysterectomy | 14.56 (1.82, 116.63) | 0.012 | 12.09 (1.63, 89.57) | 0.015 | 12.23 (1.74, 85.99) | 0.012 | 12.68 (1.63, 98.65) | 0.015 | 10.62 (3.02, 37.29) | <0.001 | 10.75 (1.58, 73.02) | 0.015 |
| GI/laparotomy | 6.10 (1.02, 36.36) | 0.047 | 5.48 (0.96, 31.31) | 0.056 | 5.24 (0.97, 28.19) | 0.054 | 6.35 (1.08, 37.28) | 0.041 | 5.70 (1.54, 21.06) | 0.009 | 5.43 (1.03, 28.74) | 0.047 |
| Urologic | 4.51 (0.45, 44.92) | 0.20 | 3.92 (0.30, 50.67) | 0.29 | 3.72 (0.41, 34.13) | 0.25 | 4.26 (0.43, 41.88) | 0.21 | 3.72 (0.24, 57.39) | 0.35 | 3.57 (0.40, 31.48) | 0.25 |

We performed similar analyses to compare the outcomes of operations with low ($\leq$2 standards) vs high ($\geq$3 standards) compliance, again calculating both odds ratios and relative risks. Again, we used both fixed- and mixed-effects models with random intercepts to account for hospital clustering; all other variable remained the same. The mixed-effects log-binomial model was unable to achieve convergence.

**Table S2:**

|  | **Fixed Effects** | | | | | | **Random Effects** | | | | | |
| --- | --- | --- | --- | --- | --- | --- | --- | --- | --- | --- | --- | --- |
|  | **Logistic Regression** | | **Modified Robust Poisson** | | **Log Binomial** | | **Logistic Regression** | | **Modified Robust Poisson** | | **Log Binomial** | |
| **Factor** | **SSI (N=2159)**  **OR (95% CI)** | **p-value** | **SSI (N=2159)**  **RR (95% CI)** | **p-value** | **SSI (N=2159)**  **RR (95% CI)** | **p-value** | **SSI (N=2159)**  **OR (95% CI)** | **p-value** | **SSI (N=2159)**  **RR (95% CI)** | **p-value** | **SSI (N=2159)**  **RR (95% CI)** | **p-value** |
| **Adherence Score** |  |  |  |  |  |  |  |  |  |  |  |  |
| 0-2 | Reference |  | Reference |  | Reference |  | Reference |  | Reference |  |  |  |
| 3-6 | 0.51 (0.27, 0.98) | 0.044 | 0.54 (0.30, 0.97) | 0.038 | 0.56 (0.31, 1.02) | 0.057 | 0.58 (0.31, 1.08) | 0.085 | 0.61 (0.39, 0.93) | 0.023 |  |  |
| **Sex** |  |  |  |  |  |  |  |  |  |  |  |  |
| Female | Reference |  | Reference |  | Reference |  | Reference |  | Reference |  |  |  |
| Male | 0.80 (0.46, 1.38) | 0.41 | 0.82 (0.51, 1.32) | 0.41 | 0.83 (0.52, 1.32) | 0.42 | 0.79 (0.46, 1.37) | 0.40 | 0.81 (0.51, 1.29) | 0.39 |  |  |
| **Age** |  |  |  |  |  |  |  |  |  |  |  |  |
| <=25 | Reference |  | Reference |  | Reference |  | Reference |  | Reference |  |  |  |
| 26-30 | 0.70 (0.42, 1.15) | 0.16 | 0.72 (0.45, 1.14) | 0.16 | 0.69 (0.44, 1.10) | 0.12 | 0.72 (0.44, 1.17) | 0.19 | 0.73 (0.45, 1.20) | 0.22 |  |  |
| 31-40 | 0.81 (0.48, 1.35) | 0.42 | 0.82 (0.51, 1.32) | 0.42 | 0.80 (0.50, 1.28) | 0.35 | 0.83 (0.50, 1.39) | 0.48 | 0.84 (0.61, 1.16) | 0.30 |  |  |
| >=41 | 0.79 (0.43, 1.46) | 0.46 | 0.80 (0.45, 1.44) | 0.46 | 0.81 (0.47, 1.39) | 0.44 | 0.81 (0.44, 1.50) | 0.51 | 0.82 (0.60, 1.12) | 0.22 |  |  |
| **Urgency** |  |  |  |  |  |  |  |  |  |  |  |  |
| Elective | Reference |  | Reference |  | Reference |  | Reference |  | Reference |  |  |  |
| Emergency | 1.10 (0.66, 1.84) | 0.71 | 1.08 (0.68, 1.72) | 0.74 | 1.05 (0.66, 1.66) | 0.84 | 1.06 (0.63, 1.77) | 0.83 | 1.04 (0.76, 1.42) | 0.81 |  |  |
| **Wound Class** |  |  |  |  |  |  |  |  |  |  |  |  |
| Clean | Reference |  | Reference |  | Reference |  | Reference |  | Reference |  |  |  |
| Clean Contaminated | 0.36 (0.09, 1.46) | 0.15 | 0.40 (0.09, 1.83) | 0.24 | 0.42 (0.12, 1.52) | 0.18 | 0.36 (0.09, 1.42) | 0.14 | 0.39 (0.08, 1.92) | 0.25 |  |  |
| Contaminated | 0.85 (0.20, 3.65) | 0.82 | 0.87 (0.19, 4.07) | 0.86 | 0.90 (0.23, 3.47) | 0.88 | 0.85 (0.20, 3.62) | 0.82 | 0.88 (0.29, 2.65) | 0.81 |  |  |
| Dirty | 2.50 (0.58, 10.90) | 0.22 | 2.10 (0.45, 9.77) | 0.35 | 2.17 (0.56, 8.39) | 0.26 | 2.48 (0.58, 10.65) | 0.22 | 2.10 (0.56, 7.90) | 0.27 |  |  |
| **Hospital** |  |  |  |  |  |  |  |  |  |  |  |  |
| 1 | Reference |  | Reference |  | Reference |  |  |  |  |  |  |  |
| 2 | 0.68 (0.31, 1.49) | 0.33 | 0.69 (0.33, 1.45) | 0.33 | 0.69 (0.34, 1.43) | 0.32 |  |  |  |  |  |  |
| 3 | 2.27 (1.08, 4.77) | 0.031 | 2.09 (1.08, 4.05) | 0.029 | 2.20 (1.13, 4.26) | 0.020 |  |  |  |  |  |  |
| 4 | 1.47 (0.67, 3.23) | 0.33 | 1.42 (0.70, 2.88) | 0.34 | 1.41 (0.69, 2.91) | 0.35 |  |  |  |  |  |  |
| 5 | 2.36 (1.17, 4.78) | 0.017 | 2.17 (1.21, 3.91) | 0.010 | 2.13 (1.14, 3.97) | 0.018 |  |  |  |  |  |  |
| **Type of operation** |  |  |  |  |  |  |  |  |  |  |  |  |
| ENT | Reference |  | Reference |  | Reference |  | Reference |  | Reference |  |  |  |
| Orthopedic | 4.76 (0.65, 34.92) | 0.12 | 4.38 (0.60, 32.24) | 0.15 | 4.19 (0.64, 27.44) | 0.14 | 4.26 (0.60, 30.49) | 0.15 | 3.95 (1.70, 9.19) | 0.001 |  |  |
| Soft tissue | 3.68 (0.82, 16.38) | 0.09 | 3.47 (0.85, 14.14) | 0.083 | 3.54 (0.85, 14.75) | 0.083 | 3.51 (0.79, 15.53) | 0.098 | 3.33 (2.09, 5.30) | <0.001 |  |  |
| Gynecologic | 4.37 (0.59, 32.28) | 0.15 | 3.99 (0.59, 26.88) | 0.16 | 4.09 (0.60, 27.78) | 0.15 | 4.05 (0.56, 29.36) | 0.167 | 3.71 (1.48, 9.27) | 0.005 |  |  |
| Vascular | 1.13 (0.11, 11.63) | 0.92 | 1.14 (0.11, 11.33) | 0.91 | 1.18 (0.12, 11.49) | 0.88 | 1.12 (0.11, 11.46) | 0.92 | 1.13 (0.17, 7.58) | 0.90 |  |  |
| Appendectomy | 5.17 (0.78, 34.24) | 0.09 | 4.76 (0.74, 30.69) | 0.10 | 4.68 (0.79, 27.77) | 0.089 | 5.41 (0.83, 35.43) | 0.08 | 4.98 (0.65, 38.03) | 0.12 |  |  |
| Cholecystectomy | 11.17 (1.27, 98.27) | 0.030 | 9.37 (1.10, 79.70) | 0.041 | 8.44 (1.14, 62.45) | 0.037 | 10.84 (1.26, 93.21) | 0.030 | 9.17 (1.52, 55.14) | 0.016 |  |  |
| Colorectal | 13.83 (2.14, 89.22) | 0.006 | 11.08 (1.74, 70.58) | 0.011 | 10.23 (1.80, 58.29) | 0.009 | 13.75 (2.15, 87.88) | 0.006 | 11.09 (3.04, 40.41) | <0.001 |  |  |
| Cesarean | 11.55 (1.72, 77.46) | 0.012 | 9.94 (1.58, 62.72) | 0.015 | 10.18 (1.70, 60.84) | 0.011 | 11.19 (1.72, 72.95) | 0.012 | 9.67 (1.80, 52.04) | 0.008 |  |  |
| Hernia | 2.44 (0.38, 15.59) | 0.35 | 2.33 (0.40, 13.46) | 0.34 | 2.31 (0.39, 13.81) | 0.36 | 2.36 (0.37, 15.07) | 0.36 | 2.26 (0.71, 7.15) | 0.17 |  |  |
| Hysterectomy | 13.25 (1.66, 106.10) | 0.015 | 11.08 (1.45, 84.72) | 0.021 | 11.21 (1.60, 78.69) | 0.015 | 11.82 (1.51, 92.39) | 0.019 | 9.96 (2.52, 39.38) | 0.001 |  |  |
| GI/laparotomy | 5.98 (1.00, 35.75) | 0.050 | 5.41 (0.92, 31.90) | 0.062 | 5.25 (0.97, 28.45) | 0.054 | 6.15 (1.04, 36.38) | 0.045 | 5.57 (1.27, 24.38) | 0.023 |  |  |
| Urologic | 4.61 (0.46, 45.90) | 0.19 | 4.01 (0.31, 51.93) | 0.29 | 3.88 (0.42, 35.68) | 0.23 | 4.33 (0.44, 42.70) | 0.21 | 3.78 (0.24, 60.48) | 0.35 |  |  |

We evaluated the relative risk of infection for each adherence score using modified robust Poisson regression controlling for sex, age, urgency, wound class, and hospital. As there were no operations with an adherence score of 0, we combine scores of 0 and 1 and used an adherence score of 6 as reference. The relative risk of infection increased non-significantly with the exception of an adherence score of 2 which demonstrated a significant increase in relative risk.

**Table S3:**

| **Factor** | **RR (95% CI)** | **p-value** |
| --- | --- | --- |
| **Adherence Score**  6 | Reference |  |
| 5 | 1.26 (0.76, 2.10) | 0.37 |
| 4 | 1.06 (0.59, 1.91) | 0.85 |
| 3 | 1.09 (0.54, 2.20) | 0.80 |
| 2 | 2.29 (1.09, 4.80) | 0.029 |
| 0-1 | 1.50 (0.43, 5.21) | 0.52 |
| **Sex**  Female  Male | Reference  0.83 (0.51, 1.34) | 0.44 |
| **Age** |  |  |
| <=25 | Reference |  |
| 26-30 | 0.72 (0.45, 1.14) | 0.16 |
| 31-40 | 0.82 (0.51, 1.32) | 0.41 |
| >=41 | 0.81 (0.46, 1.45) | 0.48 |
| **Urgency**  Elective  Emergency | Reference  1.08 (0.68, 1.72) | 0.73 |
| **Wound Class** |  |  |
| Clean | Reference |  |
| Clean Contaminated | 0.41 (0.09, 1.83) | 0.24 |
| Contaminated | 0.89 (0.20, 4.07) | 0.88 |
| Dirty | 2.17 (0.48, 9.85) | 0.32 |
| **Hospital**  1  2  3  4  5 | Reference  0.72 (0.34, 1.51)  2.16 (1.04, 4.45)  1.42 (0.69, 2.91)  2.13 (1.18, 3.83) | 0.38  0.038  0.34  0.012 |
| **Type of operation** |  |  |
| ENT | Reference |  |
| Orthopedic | 4.50 (0.61, 33.32) | 0.14 |
| Soft tissue | 3.66 (0.89, 15.01) | 0.072 |
| Gynecologic | 3.98 (0.59, 26.70) | 0.15 |
| Vascular | 1.23 (0.12, 12.48) | 0.86 |
| Appendectomy | 4.76 (0.76, 29.94) | 0.096 |
| Cholecystectomy | 9.36 (1.13, 77.56) | 0.038 |
| Colorectal | 11.12 (1.79, 69.02) | 0.010 |
| Cesarean | 9.40 (1.53, 57.62) | 0.015 |
| Hernia | 2.31 (0.40, 13.22) | 0.35 |
| Hysterectomy | 11.04 (1.48, 82.52) | 0.019 |
| GI/laparotomy | 5.51 (0.96, 31.60) | 0.056 |
| Urologic | 3.92 (0.31, 48.89) | 0.29 |

We assessed the relative risk of postoperative infection and exponentiated linear regression coefficient of length of stay based on timing of process improvements after adjusting for sex, age, urgency, wound class, and hospital.

**Table S4:**

| **Factor** | **SSI (N=2159)**  **RR (95% CI)** | **p-value*** | **Length of Stay (N=785)**  **Ratio^#^ (95% CI)** | **p-value ^#^** |
| --- | --- | --- | --- | --- |
| **Infection** |  |  |  |  |
| Baseline | Reference |  |  |  |
| Post-Implementation | 0.65 (0.43, 0.99) | 0.043 | 0.99 (0.90, 1.09) | 0.8867 |
| **Sex** |  |  |  |  |
| Female | Reference |  |  |  |
| Male | 0.83 (0.52, 1.32) | 0.43 | 1.06 (0.94, 1.20) | 0.3105 |
| **Age** |  |  |  |  |
| <=25 | Reference |  |  |  |
| 26-30 | 0.72 (0.45, 1.15) | 0.17 | 0.96 (0.89, 1.04) | 0.3059 |
| 31-40 | 0.81 (0.50, 1.30) | 0.38 | 1.06 (0.97, 1.16) | 0.1721 |
| >=41 | 0.82 (0.46, 1.47) | 0.51 | 1.31 (1.16, 1.48) | <.0001 |
| **Urgency** |  |  |  |  |
| Elective | Reference |  |  |  |
| Emergency | 1.10 (0.70, 1.74) | 0.68 | 0.92 (0.83, 1.02) | 0.1137 |
| **Wound Class** |  |  |  |  |
| Clean | Reference |  |  |  |
| Clean Contaminated | 0.38 (0.08, 1.68) | 0.20 | 0.78 (0.60, 1.00) | 0.0522 |
| Contaminated | 0.84 (0.18, 3.87) | 0.83 | 0.80 (0.58, 1.09) | 0.1504 |
| Dirty | 2.01 (0.44, 9.22) | 0.37 | 1.08 (0.72, 1.62) | 0.7120 |
| **Type of operation** |  |  |  |  |
| ENT | Reference |  | Reference |  |
| Orthopedic | 4.44 (0.63, 31.19) | 0.13 | - |  |
| Soft tissue | 3.72 (0.92, 15.09) | 0.066 | 2.22 (1.55, 3.17) | <.0001 |
| Gynecologic | 4.36 (0.62, 30.44) | 0.14 | 1.43 (1.10, 1.86) | 0.0074 |
| Vascular | 1.22 (0.12, 12.27) | 0.87 | - |  |
| Appendectomy | 4.73 (0.76, 29.63) | 0.10 | 1.09 (0.78, 1.52) | 0.6276 |
| Cholecystectomy | 9.89 (1.20, 81.43) | 0.033 | - |  |
| Colorectal | 10.93 (1.76, 67.92) | 0.010 | 2.49 (1.76, 3.53) | <.0001 |
| Cesarean | 11.09 (1.82, 67.74) | 0.009 | 1.32 (0.98, 1.79) | 0.0685 |
| Hernia | 2.31 (0.40, 13.34) | 0.35 | 0.95 (0.74, 1.22) | 0.7061 |
| Hysterectomy | 12.09 (1.63, 89.57) | 0.015 | 1.91 (1.33, 2.75) | 0.0005 |
| GI/laparotomy | 5.48 (0.96, 31.31) | 0.056 | 1.75 (1.30, 2.37) | 0.0003 |
| Urologic | 3.92 (0.30, 50.67) | 0.29 | 1.36 (0.96, 1.92) | 0.0865 |

(Relative risks by hospital are not shown)

* Modified robust Poisson regression

# Exponentiated linear regression; exponentiated coefficients represent ratios relative to the reference group.

We assessed the relative risk of postoperative infection and exponentiated linear regression coefficient of length of stay, based on adherence to six critical standards in infection prevention after adjusting for sex, age, urgency, wound class, and hospital.

**Table S5**:

| **Factor** | **SSI (N=2159)**  **RR (95% CI)** | **p-value*** | **Length of Stay (N=784)**  **Ratio^#^ (95% CI)** | **p-value ^#^** |
| --- | --- | --- | --- | --- |
| **Adherence Score** |  |  |  |  |
| 0-2 | Reference |  | Reference |  |
| 3-6 | 0.54 (0.30, 0.97) | 0.038 | 0.98 (0.89, 1.07) | 0.65 |
| **Sex** |  |  |  |  |
| Female | Reference |  | Reference |  |
| Male | 0.82 (0.51, 1.32) | 0.41 | 1.06 (0.94, 1.19) | 0.32 |
| **Age** |  |  |  |  |
| <=25 | Reference |  | Reference |  |
| 26-30 | 0.72 (0.45, 1.14) | 0.16 | 0.96 (0.89, 1.04) | 0.30 |
| 31-40 | 0.82 (0.51, 1.32) | 0.42 | 1.06 (0.97, 1.16) | 0.17 |
| >=41 | 0.80 (0.45, 1.44) | 0.46 | 1.31 (1.16, 1.48) | <0.001 |
| **Urgency** |  |  |  |  |
| Elective | Reference |  | Reference |  |
| Emergency | 1.08 (0.68, 1.72) | 0.74 | 0.92 (0.83, 1.02) | 0.12 |
| **Wound Class** |  |  |  |  |
| Clean | Reference |  | Reference |  |
| Clean Contaminated | 0.40 (0.09, 1.83) | 0.24 | 0.78 (0.61, 1.00) | 0.052 |
| Contaminated | 0.87 (0.19, 4.07) | 0.86 | 0.80 (0.58, 1.09) | 0.16 |
| Dirty | 2.10 (0.45, 9.77) | 0.35 | 1.08 (0.72, 1.62) | 0.70 |
| **Type of operation** |  |  |  |  |
| ENT | Reference |  | Reference |  |
| Orthopedic | 4.38 (0.60, 32.24) | 0.15 | - |  |
| Soft tissue | 3.47 (0.85, 14.14) | 0.083 | 2.21 (1.54, 3.16) | <0.001 |
| Gynecologic | 3.99 (0.59, 26.88) | 0.16 | 1.43 (1.10, 1.86) | 0.008 |
| Vascular | 1.14 (0.11, 11.33) | 0.91 | - |  |
| Appendectomy | 4.76 (0.74, 30.69) | 0.10 | 1.09 (0.78, 1.52) | 0.63 |
| Cholecystectomy | 9.37 (1.10, 79.70) | 0.041 | - |  |
| Colorectal | 11.08 (1.74, 70.58) | 0.011 | 2.49 (1.76, 3.52) | <0.001 |
| Cesarean | 9.94 (1.58, 62.72) | 0.015 | 1.32 (0.98, 1.79) | 0.07 |
| Hernia | 2.33 (0.40, 13.46) | 0.34 | 0.96 (0.74, 1.23) | 0.72 |
| Hysterectomy | 11.08 (1.45, 84.72) | 0.021 | 1.91 (1.33, 2.75) | <0.001 |
| GI/laparotomy | 5.41 (0.92, 31.90) | 0.062 | 1.75 (1.30, 2.37) | <0.001 |
| Urologic | 4.01 (0.31, 51.93) | 0.29 | 1.36 (0.96, 1.93) | 0.08 |

(Relative risks by hospital are not shown)

* Modified robust Poisson regression

# Exponentiated linear regression; exponentiated coefficients represent ratios relative to the reference group.

Adherence to best practices improved at all facilities as noted in the below radar plots; p-values calculated using Fisher’s exact test:

Hospital 1:

Hospital 2:

Hospital 3:

Hospital 4:

Hospital 5:
